# The Influence of Polymer Composition on the Hydrolytic and Enzymatic Degradation of Polyesters and Their Block Copolymers with PDMAEMA

**DOI:** 10.3390/ma14133636

**Published:** 2021-06-29

**Authors:** Maria Kupczak, Anna Mielańczyk, Dorota Neugebauer

**Affiliations:** Department of Physical Chemistry and Technology of Polymers, Faculty of Chemistry, Silesian University of Technology, ks. Marcina Strzody 9, 44-100 Gliwice, Poland; maria.kupczak@polsl.pl

**Keywords:** (bio)degradable polyesters, block copolymers, hydrolytic degradation, enzymatic degradation, PDMAEMA

## Abstract

Well-defined, semi-degradable polyester/polymethacrylate block copolymers, based on ε-caprolactone (CL), d,l-lactide (DLLA), glycolide (GA) and *N*,*N′*-dimethylaminoethyl methacrylate (DMAEMA), were synthesized by ring-opening polymerization (ROP) and atom transfer radical polymerization. Comprehensive degradation studies of poly(ε-caprolactone)-*block*-poly(*N*,*N′*-dimethylaminoethyl methacrylate) (PCL-*b*-PDMAEMA) on hydrolytic degradation and enzymatic degradation were performed, and those results were compared with the corresponding aliphatic polyester (PCL). The solution pH did not affect the hydrolytic degradation rate of PCL (a 3% M_n_ loss after six weeks). The presence of a PDMAEMA component in the copolymer chain increased the hydrolysis rates and depended on the solution pH, as PCL-*b*-PDMAEMA degraded faster in an acidic environment (36% M_n_ loss determined) than in a slightly alkaline environment (27% M_n_ loss). Enzymatic degradation of PCL-*b*-PDMAEMA, poly(d,l-lactide)-*block*-poly(*N*,*N′*-dimethylaminoethyl methacrylate) (PLA-*b*-PDMAEMA) and poly(lactide-*co*-glycolide-*co*-ε-caprolactone)-*block*-poly(*N*,*N′*-dimethylaminoethyl methacrylate) (PLGC-*b*-PDMAEMA) and the corresponding aliphatic polyesters (PCL, PLA and PLGC) was performed by Novozyme 435. In enzymatic degradation, PLGC degraded almost completely after eleven days. For polyester-*b*-PDMAEMA copolymers, enzymatic degradation primarily involved the ester bonds in PDMAEMA side chains, and the rate of polyester degradation decreased with the increase in the chain length of PDMAEMA. Amphiphilic copolymers might be used for biomaterials with long-term or midterm applications such as nanoscale drug delivery systems with tunable degradation kinetics.

## 1. Introduction

Polymers and polymeric materials are widely used in every area of everyday life, including home appliances, housing, packaging, electronics and construction materials in the automotive and construction industries [1,2]. The increased interest in polymeric materials and their production has accelerated the development of the economy; however, it has also resulted in a significant waste production increase [3,4]. These wastes, in turn, pose an environmental threat due to improper waste management, long decomposition times and potentially harmful decomposition products. Biodegradable polymers provide an opportunity to produce environmentally friendly materials with properties similar to those of conventional polymers [5,6,7]. In addition, biodegradable polymers, due to their unique properties, which are biocompatibility in relation to living tissues, biodegradability and non-immunogenicity, are used, among others, in the biomedical field. They are used as biomaterials for various applications, which include absorbable sutures, bone screws and plates, stents, drug carriers and tissue engineering scaffolds. The advantages of biodegradable polymers as biomedical materials include: no need to remove polymers from the body after fulfilling their role; the long-term toxicity and inflammation caused by low-molecular weight degradation are unlikely to occur since these products can be metabolized or excreted from the body; and sustained release of drugs [8].

Among the different types of biodegradable polymers, aliphatic polyesters comprising homo- and copolymers of glycolide, dilactide and ε-caprolactone are the most studied [9] because they are synthesized by ring-opening polymerization (ROP), which controls their molecular weight, composition and topology. These features are known to be crucial for the degradation rate of (co)polyesters and create opportunities to obtain materials with specific lifetimes.

Although myriad literature reports focus on the degradation of poly(ε-caprolactone) (PCL) [10], poly(DL-lactide) (PDLLA) [11,12] and poly(lactide-*co*-glycolide) (PLGA) [13,14,15], another emerging polymer group features copolymers with different types of degradable and non-degradable blocks. Recently, water-soluble polyethylene glycol (PEG), polypropylene glycol (PPG) and polyvinylpyrrolidone (PVP) have modulated polyester biodegradability by adjusting the hydrophilicity. It has been reported that PLA-PEG [16,17,18], PLA-PEG-PPG-PEG [19], PCL-PEG-PCL [20,21], PEG-PCL-PLA [22] and PLA-PVP-PLA [23] copolymers all have better hydrophilicity and faster degradation rates in comparison to PLA and PCL homopolymers. Another important feature of PEG, PPG and PVP is that they are electrically neutral at all pH values. Furthermore, to the best of our knowledge, there are no literature reports concerning the degradation of PCL, PLA and PLGC copolymers with polycations such as poly(*N*,*N’*-dimethylaminoethyl methacrylate) PDMAEMA in a phosphate buffered saline PBS solution or by using Novozyme 435.

Our previous studies involved comprehensive degradation studies on enzymatic degradation using lipase from Pseudomonas cepacia [24]. Herein, we performed a degradation study of PCL-*b*-PDMAEMA, PLA-*b*-PDMAEMA and PLGC-*b*-PDMAEMA copolymers under simulated physiological conditions, namely, hydrolytic degradation in PBS at different pHs, and enzymatic degradation using Novozyme 435. Furthermore, we conducted the degradation of three representative aliphatic polyesters (i.e., PCL, PLA, PLGC) under identical conditions to compare those results and establish preliminary structure−degradation relationships. In the future, the obtained block copolymers are to be used in drug delivery systems with regulated degradation kinetics.

## 2. Materials and Methods

### 2.1. Materials

Anisole (Alfa Aesar, 99%, Warsaw, Poland), methanol (Chempur, p. a., Piekary Śląskie, Poland), 2-hydroxyethyl 2-bromoisobutyrate (HEBiB, Aldrich 95%, Poznań, Poland), *N*,*N’*-dimethylaminoethyl methacrylate (DMAEMA, Aldrich, 98%, Poznań, Poland) and triethylene glycol monomethyl ether (MTEG, Aldrich 95%, Poznań, Poland) were stored over molecular sieves in a freezer under nitrogen. Toluene and ε-caprolactone (CL, Alfa Aesar, 99%, Warsaw, Poland) were distilled prior to use and stored over molecular sieves. Glycolide (GL, Aldrich, 99%, Poznań, Poland), 3,6-dimethyl-1,4-dioxane-2,5-dione (D,L-lactide, Aldrich, 99%, Poznań, Poland), *N*, *N*, *N’*, *N’’*, *N’’*-pentamethyldiethylenetriamine (PMDETA, Aldrich, 99%, Poznań, Poland) and sodium azide (Acros, 99 %, Geel, Belgium) were used as received. Copper(I) chloride (CuCl, Fluka, 98%, Steinheim, Germany) was purified by stirring in glacial acetic acid, followed by filtration and washings with ethanol and diethyl ether, and dried under vacuum. Tin(II) bis(2-ethylhexanoate) (Sn(Oct)_2_, Alfa Aesar, 96%, Warsaw, Poland) was distilled prior to use. Tetrahydrofuran (THF, Aldrich, HPLC, Poznań, Poland), n-heptane (Chempur, 99%, Piekary Śląskie, Poland) and methylene chloride (CH_2_Cl_2_, Chempur, 99%, Piekary Śląskie, Poland) were used as received. Novozyme 435 was purchased from STREM Chemicals, Inc. (Kehl, Germany).

The syntheses of polymers used in these experiments are described in the Supplementary Materials.

### 2.2. Hydrolytic Degradation

Hydrolytic degradation was conducted on the selected polymers (PCL2 and 1PCL2-*b*-PDMAEMA) in PBS solution at pH levels of 5.0 and 7.4 with the addition of sodium azide (23 μM), which protected the buffer against microbial growth. The polymer samples (dry powder insoluble in PBS) were weighed (8 mg ± 0.5 mg) into vials and filled with the PBS solution. Additionally, vials containing PBS only, at pHs of 7.4 and 5.0, were prepared and marked as zero tests. All vials were capped, secured with Teflon tape and placed in a shaker incubator (ES-80, Grant Instruments LTD, Royston, UK) at 37 °C. At predetermined time intervals, samples were removed from the incubator, frizzed and lyophilized. The degree of polymer degradation was calculated based on the average sample weight loss as determined by nuclear magnetic resonance (^1^H NMR) and size exclusion chromatography (SEC) analyses. Samples were analyzed in triplicate.

### 2.3. Enzymatic Degradation

Enzymatic degradation was conducted on selected polyesters (PCL1, PLA1, PLGC1) and copolymers (2PCL2-*b*-PDMAEMA, PLA2-*b*-PDMAEMA, PLGC2-*b*-PDMAEMA). First, approximately 20 ± 0.5 mg of each polymeric sample was weighed into glass vials. After dissolution of polymeric samples in toluene (10 mL), 10 mg of Novozyme 435 was added to each vial. The vials and their contents were sealed and placed on a shaker at room temperature (25 ± 2 °C). All vials remained under these conditions for three weeks, during which time, 500 μL aliquots of each sample solution were taken at specified time intervals. The degree of polymer degradation was calculated based on the average sample weight loss as determined by SEC analyses. Samples were analyzed in triplicate.

## 3. Characterization

### 3.1. Nuclear Magnetic Resonance (^1^H NMR)

^1^H NMR spectra of the synthesized polymer solutions in CDCl_3_ were collected on a Varian Inova 600 MHz spectrometer (Palo Alto, CA, USA) at 25 °C using TMS as an internal standard.

### 3.2. Size Exclusion Chromatography (SEC)

Molecular weights and dispersity (Ð) indices were determined using size exclusion chromatography (SEC, 1100 Agilent 1260 Infinity) (Agilent Technologies, Santa Clara, CA, USA) equipped with an isocratic pump, autosampler, degasser, a thermostatic box for columns and a differential refractometer MDS RI Detector. Addon Rev. B.01.02 data analysis software (Agilent Technologies) was used for data collection and processing. The SEC-calculated molecular weight was based on a calibration using linear polystyrene standards (580–300,000 g/mol). A pre-column guard, 5 µm 50 × 7.5 mm, and two columns, PLGel 5 µm MIXED-C 300 × 7.5 mm and PLGel 5 µm MIXED-D 300 × 7.5 mm, were used for separation. The measurements were obtained using THF (HPLC grade) as the solvent at 40 °C and a flow rate of 0.8 mL/min.

## 4. Results and Discussion

### 4.1. Polymer Characterization before Degradation

Linear polymers: PCL, PLA and PLGC were obtained by ROP. Amphiphilic block copolymers composed of aliphatic polyesters and PDMAEMA were obtained by combining polymerization techniques such as ATRP and ROP (Scheme S1). These polyesters and amphiphilic polyester-*b*-PDMAEMA copolymers were examined for their susceptibility to undergo hydrolytic and enzymatic degradation in environments at certain pH levels.

Table 1 and Table 2 show the characterization of the synthesized polymers. The degree of cyclic (di)ester polymerization (DP) and the theoretical number-average molecular weights (M_n,theo_) of these polyesters were calculated using signals belonging to an initiator and appropriate repeating unit present in the ^1^H NMR spectra. Figure S1 presents the spectra of the synthesized copolymers with characteristic peaks belonging to PDMAEMA and a proper polyester segment.

In addition, ATR-FTIR analysis confirmed the polymer structures (Figure S2). Five major absorption bands were observed in the spectra, indicating the presence of specific functional groups. The most intense signal, characteristic of the polymers, was the C=O stretching vibration from 1690 to 1800 cm^−1^. In this area, at PLA2-*b*-PDMAEMA, two overlapping absorption bands corresponding to C=O stretching vibrations are visible—one from the main polyester chain and the other from the ester bond found in the PDMAEMA structure. Another characteristic absorption band corresponding to C–O stretching vibrations from 1050 to 1300 cm^−1^ was also observed. All spectra have C–H stretching vibrations from -CH2- methyl groups from 2900 to 3100 cm^−1^. Furthermore, there were also C–H deformation absorption bands for the -CH2- groups at 1450–1500 cm^−1^. Spectra of polyester-b-PDMAEMA copolymers possessed additional peaks that corresponded to C–N and C–H stretching vibrations due to the -N(CH3)2 group from 1180 to 1360 cm^−1^ and 2755 to 2850 cm^−1^, respectively.

**Figure 1:**
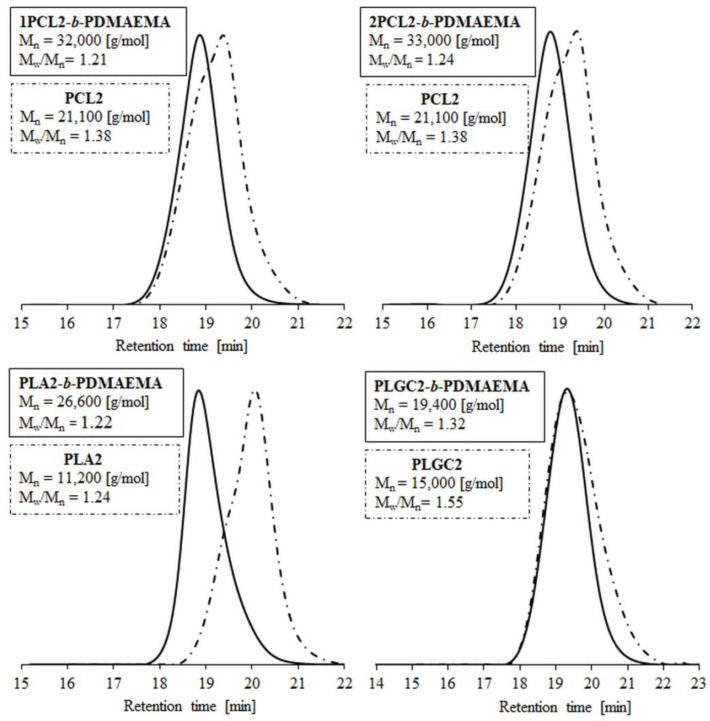
SEC traces of polyesters and their block polymers with PDMAEMA.

The SEC traces of polyester macroinitiators and corresponding block polymers are shown in Figure 1. The low dispersity values (M_w_/M_n_) (1.2–1.4) indicate sustained control over the polymerization process. The number-average molecular weights, as determined by SEC (M_n;SEC_), ranged from 19,400 to 33,300 g/mol and were higher than the theoretical ones (M_n;theoretical._ = 15,000–20,000 g/mol). The hydrodynamic volume differences in the tested samples and the standard resulted from their chemical natures (PS is hydrophobic and PDMAEMA/polyester are amphiphilic).

### 4.2. Hydrolytic Degradation

**Figure 2:**
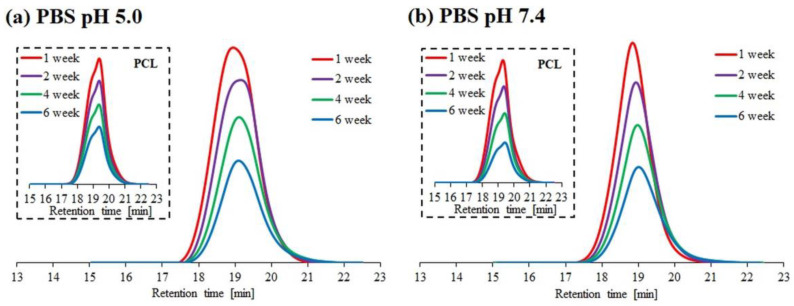
SEC traces obtained for hydrolytic degradation of copolymer 1PCL2-*b*-PDMAEMA at pH 5.0 (**a**) and pH 7.4 (**b**). SEC traces for the corresponding macroinitiator PCL2 are placed in brackets.

**Figure 3:**
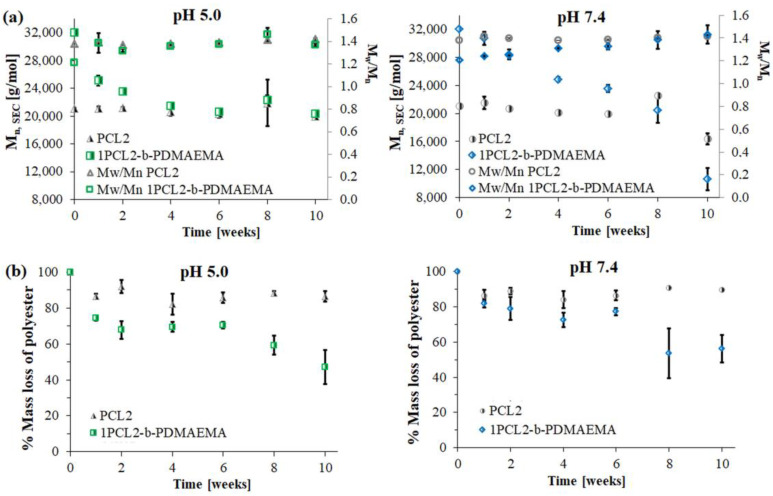
Dependence of M_n_, changes over time for 1PCL2-*b*-PDMAEMA and PCL2 based on SEC (**a**) and percentage weight loss of PCL block based on ^1^H NMR (**b**) analyses.

The results of hydrolytic degradation for the block copolymer 1PCL2-b-PDMAEMA and the corresponding macroinitiator PCL2 are presented as SEC traces (Figure 2) and graphs showing the dependence of molecular weight loss: M_n,SEC_ (based on SEC analyses), and M_n,theo_ (based on 1H NMR analyses), over time (Figure 3).

Similar to previous literature reports regarding PCL degradation [25,26], the SEC PCL2 macroinitiator analysis results show a slight molecular weight decrease at both pH 5.0 and 7.4. This was due to the high crystallinity of the polymer and low density of the ester groups on its backbone as compared to PLA. For 1PCL2-*b*-PDMAEMA, SEC analysis showed a gradual decrease in M_n,SEC_ at pH 7.4, and an abrupt decrease in Mn at pH 5.0 (Figure 2a). Four weeks after beginning the experiment, the M_n,SEC_ value for 1PCL2-*b*-PDMAEMA at pH 5.0 dropped from 32,000 to 22,000 g/mol (Figure 3a). The rate of the molecular weight loss stalled, and M_n,SEC_ values hovered around 21,000 g/mol from weeks two to four and ~20,000 g/mol after ten weeks. In a slightly alkaline medium (pH 7.4), based on data obtained from SEC, a linear decrease in the M_n,SEC_ value of the 1PCL2-b-PDMAEMA samples was observed. After ten weeks, the M_n_ decreased from 32,000 to 10,600 g/mol.

Figure 3b presents the molecular weight loss percentage of the PCL fraction calculated from ^1^H NMR spectra of samples taken at predetermined times of hydrolytic degradation. Regardless of the solution pH, samples containing the PCL2 homopolymer did not show weight losses above 10%, regardless of the hydrolytic degradation time, whereas ^1^H NMR results for the 1PCL2-*b*-PDMAEMA samples showed a slightly higher average DP_CL_ decrease in an acidic pH than an alkaline solution (DP_CL_ = 53 at pH 5.0, DP_CL_ = 63 at pH 7.4).

The discrepancy between results obtained from SEC and ^1^H NMR was related to the limitation of the SEC method and partial self-catalyzed cleavage of *N,N*-dimethyethylamine from PDMAEMA side chains [27]. Under acidic conditions, the amino groups protonate, resulting in the formation of quaternary ammonium cations, which increases the solubility of the polymer. This may result in an increase in the rate of hydrolysis of the copolymer at acidic pH due to the increased water uptake [28]. Nevertheless, the weight loss of copolymers in PBS indicates that samples with PDMAEMA increased the water uptake by the polymer films, which resulted in a faster degradation rate. These results are in good agreement with those obtained by Little et al., where the addition of hydrophilic poly(aspartic acid-*co*-lactide) (PAL) improved the PCL degradation rate. Samples containing an 8 wt.% mol fraction of hydrophilic PAL had a 20% weight loss after seven months [26]. Based on the erosion model developed by Burkersroda et al., PCL2 as well as 1PCL2-*b*-PDMAEMA most likely underwent bulk erosion [29]. Although samples were in the form of powder dispersed in PBS solution, the addition of the hydrophilic PDMAEMA block enhanced the diffusion of water into the bulk and enabled polyester chain scission.

### 4.3. Enzymatic Degradation

**Figure 4:**
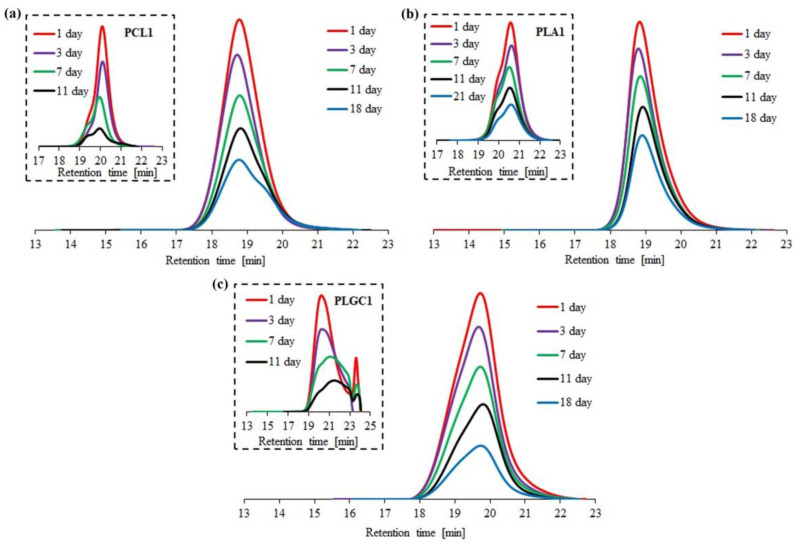
SEC traces obtained for enzymatic degradation of copolymer 2PCL2-*b*-PDMAEMA (**a**), copolymer PLA2-*b*-PDMAEMA (**b**) and copolymer PLGC2-*b*-PDMAEMA (**c**). SEC traces for the corresponding aliphatic polyesters are placed in brackets.

Enzymatic degradation with Novozyme 435 was performed for three block polymers (2PCL2-*b*-PDMAEMA, PLA2-*b*-PDMAEMA, PLGC2-*b*-PDMAEMA) and the corresponding aliphatic polyesters. Based on SEC traces, the PLGC terpolymer almost completely degraded after eleven days (Figure 4c).

**Figure 5:**
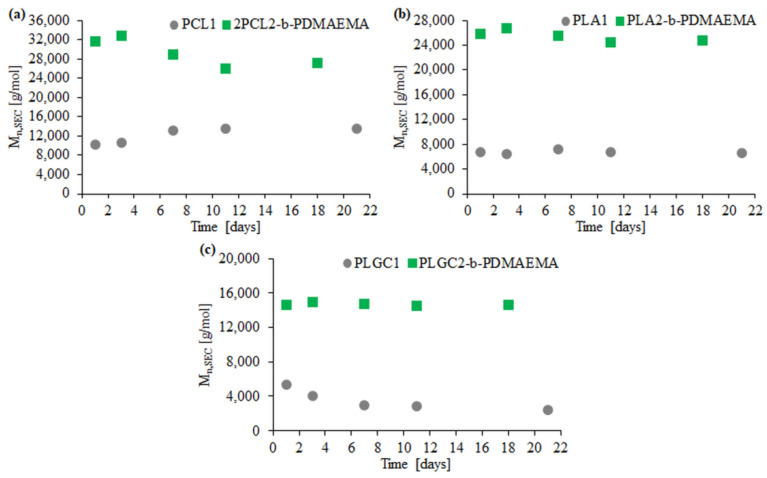
Graphs of Mn loss dependence of the polyester block over time for 2PCL2-*b*-PDMAEMA (**a**), PLA2-*b*-PDMAEMA (**b**) and PLGC2-*b*-PDMAEMA (**c**) based on SEC.

For PCL1, PLA1 and PLGC1, degradation clearly progressed. According to SEC eluograms, signals from PCL1 and PLGC1 gradually weakened and, after 18 days, almost completely disappeared (Figure 4a,c). Additionally, in the case of PCL1, no shift towards higher retention time values was observed. Thus, a decrease in the molecular weight of PCL1 was not observed, but instead, a slight increase (Figure 5a). Moreover, a steep decline in M_n,SEC_ of PLA1, PLGC1 and PLGC2-*b*-PDMAEMA, in the first 24 h, was observed. The obtained results suggest that degradation of PCL1, PLGC1 and PLGC2-*b*-PDMAEMA using Novozyme 435 in toluene proceeded by random chain scission on the polymer backbone [30], whereas PLA1 degradation occurred via chain-end and random chain scission simultaneously. The profile of weight loss of 2PCL2-*b*-PDMAEMA within 18 days suggests chain-end scission rather than the random chain scission process. For PLA2-*b*-PDMAEMA degradation, the slight molecular weight decrease observed on SEC chromatograms resulted from cleavage of a few ester bonds on the PDMAEMA side chains. Further, no molecular weight decrease was observed, as shown in Figure 5b. According to the hydrophilic fraction content in block copolymers, the degradation seems to be inhibited with the increase in the length of the PDMAEMA block. This can be explained by the interactions between the hydrophilic PDMAEMA and the immobilized lipase enzyme. PDMAEMA either blocks the active sites or deactivates the protein [31,32].

## 5. Conclusions

Using the bifunctional initiator 2-hydroxyethyl 2-bromoisobutyrate afforded several well-defined amphiphilic polymers that contained biodegradable polyester blocks and thermo/pH-sensitive polymethacrylate blocks in their structures. Hydrolytic degradation studies showed the sample containing the PCL homopolymer degraded very slowly. PCL, regardless of the pH, degraded at the same rate—a 3% M_n_ loss after six weeks. On the other hand, the presence of PDMAEMA in the copolymer chain resulted in different decomposition rates, depending on the pH. 1PCL2-*b*-PDMAEMA degraded faster in an acid environment (36% M_n_ loss determined after 10 weeks) than in a slightly alkaline environment (27% M_n_ loss after 10 weeks). This suggests the polycationic PDMAEMA block in the acidic environment protonated, formed quaternary ammonium groups that repelled each other and made the copolymer more susceptible to hydrolytic degradation due to its higher hydrophilicity. In enzymatic degradation, PLGC1 degraded the fastest and confirmed previous reports that showed the addition of LA and GA repeating units increased the degradation rate of the aliphatic polyester. However, for polyester-*b*-PDMAEMA copolymers, the enzymatic degradation rate decreased with the increase in the length of the PDMAEMA block. Although the addition of the PDMAEMA block to the PCL increased the rate of polyester hydrolysis in PBS, the rate of bulk erosion in the enzymatic degradation of the polyester was hampered by the presence of the hydrophilic polymethacrylate. The obtained results show that the time of polyester degradation can be controlled by the addition of a polyamine fraction with a proper length.

## Figures and Tables

**Table 1:** Characterization of aliphatic polyesters.

Polymer	Initiator	DP_CL_ ^a^	DP_LA_ ^a^	DP_GA_ ^a^	M_n,NMR_ ^a^ (g/mol)	M_n,SEC_ ^b^ (g/mol)	M_w_/M_n_ ^b^
PCL1	MTEG	82	-	-	9400	11,300	1.15
PLA1		-	204	-	14,700	13,400	1.17
PLGC1		22	125	30	13,300	13,300	1.49
PCL2	HEBiB	112	-	-	12,800	21,100	1.38
PLA2		-	112	-	8100	11,200	1.24
PLGC2		17	94	24	10,100	15,000	1.55

Where [M]_0_:[I]_0_:[Sn(Oct)_2_]_0_ = 100:1:0.1, except PLA2, where: [M]_0_:[I]_0_:[Sn(Oct)_2_]_0_ = 50:1:0.1. ^a^ Degree of polymerization was determined by ^1^H NMR measurements. ^b^ Determined by SEC measurements on the basis of polystyrene standards.

**Table 2:** Characterization of polyester-*b*-PDMAEMA linear block copolymers.

Polymer	DP_Polyester_ ^a^/DP_DMAEMA_ ^b^	F_hydrophilic_ ^c^	M_n,theo_ ^d^ (g/mol)	M_n,SEC_ ^e^ (g/mol)	M_w_/M_n_ ^e^
1PCL2-*b*-PDMAEMA	112/44	0.32	19,700	32,000	1.21
2PCL2-*b*-PDMAEMA	112/40	0.26	19,100	33,000	1.24
PLA2-*b*-PDMAEMA	112/76	0.40	20,000	26,600	1.22
PLGC-*b*-PDMAEMA	135/31	0.19	15,000	19,400	1.32

Where [DMEAME]_0_:[MI]_0_:[CuCl]_0_:[L]_0_ = 100:1:1:1, anisole 75% *v*/*v* mon. ^a^ Degree of polymerization was determined by ^1^H NMR measurements. ^b^ Degree of polymerization was determined by GC measurements. ^c^ Mole fraction of hydrophilic repeating units (F_hydrophilic_) was calculated based on the equation: F_hydrophilic_ = DP_DMAEMA_/(DP_Polyester_ + DP_DMAEMA_). ^d^ Theoretical number-average molecular weight (M_n,theo_) was calculated based on the equation: M_n,theo_ = DP_Polyester_ + DP_DMAEMA_. ^e^ Determined by SEC measurements based on polystyrene standards.

## Data Availability

The data presented in this study are available on request from the corresponding author.

## Supplementary Materials

The following are available online at https://www.mdpi.com/article/10.3390/ma14133636/s1, Scheme S1: Schematic representation for the synthesis of linear (A) and amphiphilic block (B) polymers, Figure S1: 1H NMR (600 MHz, CDCl3) spectra of obtained copolymers before degradation, Figure S2: ATR-FTIR spectra of obtained polymers before degradation.
