# Supplementary material for: The Influence of Polymer Composition on the Hydrolytic and Enzymatic Degradation of Polyesters and Their Block Copolymers with PDMAEMA
Source: Materials (Basel). 2021 Jun 29;14(13):3636. doi: 10.3390/ma14133636 (PMC8269683; doi:10.3390/ma14133636)
Supplement: Supplementary file 1 [file materials-14-03636-s001.zip › materials-1238862-supplementary.pdf]

materials

MDPI

Article

The Influence of Polymer Composition on the Hydrolytic and Enzymatic Degradation of Polyesters and Their Block Copolymers with PDMAEMA

Maria Kupczak, Anna Mielańczyk * and Dorota Neugebauer *

Department of Physical Chemistry and Technology of Polymers, Faculty of Chemistry, Silesian University of Technology, ks. Marcina Strzody 9, 44-100 Gliwice, Poland; maria.kupczak@polsl.pl
* Correspondence: anna.mielanczyk@polsl.pl (A.M.); dorota.neugebauer@polsl.pl (D.N.)

1. Synthesis of Aliphatic Polyesters and polyster-*b*-PDMAEMA Copolymers

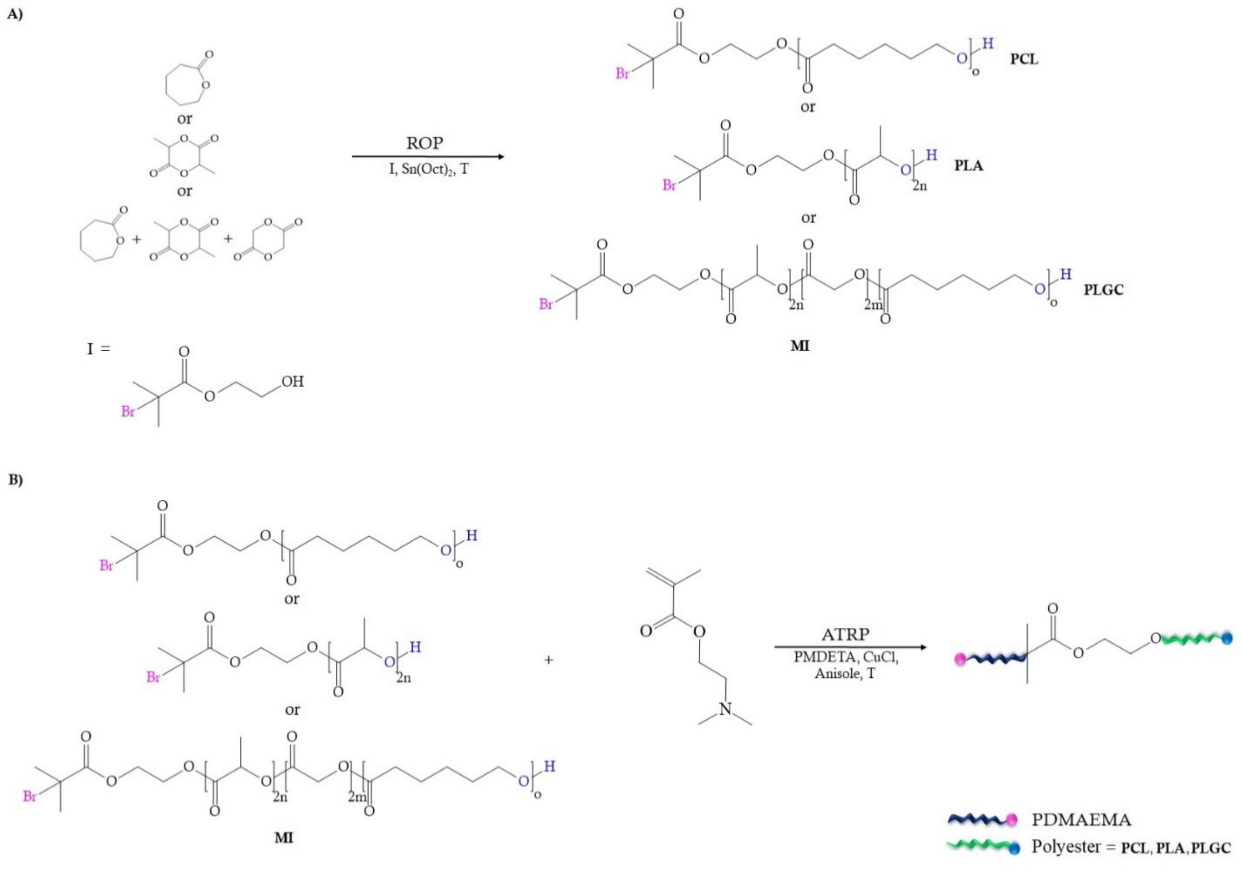

Scheme S1. Schematic representation for the synthesis of linear (A) and amphiphilic block (B) polymers**.**

1.1. Synthesis of Polyester Macroinitiator (MI1)

Macroinitiators of polyester type were synthesized via coordination-insertion ring-opening polymerization.

Prior to the synthesis, the Schlenk reactor was subjected to silanization. For this purpose, 6% chlorotrimethylsilane solution in toluene was prepared in a Schlenk reactor, and left for 24 hours. After 24 hours, the solution was poured out of the reactor, which was then immersed into a hot oil bath (120 ° C) and heated under vacuum for 2 hours. After cooling the reactor to room temperature, CL (27 mmol, 3 mL) and HEBIB (0.27 mmol, 39 µL) were introduced under argon. Then the mixture was subjected to the three freeze-

pump-thaw cycles. Next, the appropriate amount of ROP catalyst was introduced into the reactor (150 μL, 6% solution of $Sn(Oct)_2$ in toluene). The polymerization process was allowed to continue for 0.5 hour at 130 °C. The reaction mixture was diluted with dichloromethane, and then the polymer was precipitated in methanol. The crude product was redissolved in dichloromethane and re-precipitated in methanol. The resulting product was transferred to a previously weighed vial and dried to constant weight. Yield: 86%, $M_{n,theo}$ = 12800 g/mol, $M_{n,SEC}$ = 21100 g/mol, Đ = 1.38).

1.2. Synthesis of polyester-b-PDMAEMA (Example for I) via ATRP

Macroinitiator (PCL, 50,33 mg; 0.05 mmol/1 initiating site), DMAEMA (0.8 mL; 5 mmol), PMDETA (10 μL; 0.05 mmol), and anisole (0.6 mL; 75 vol% of monomer) were placed in a Schlenk flask and then degassed by three freeze-pump-thaw cycles. After that CuCl (4.7 mg; 0.05 mmol), was added, and the reaction flask was immersed in an oil bath at 45 °C. The reaction was stopped after 2 hours by exposing the reaction mixture to air. After dilution of reaction mixture with CH_2Cl_2, the CuCl/PMDETA complex was removed by passing through basic alumina column. The organic solvent was evaporated and the final product was precipitated in n-heptan and dried to constant mass. Yield: 58%, $M_{n,theo}$ = 19100 g/mol, $M_{n,SEC}$ = 33000 g/mol, Đ = 1.24).

2. Characterization of Polyesters and polyester-*b*-PDMAEMA Copolymers before Degradation

2.1. Gas Chromatography (GC)

Conversion of DMAEMA was determined by gas chromatography. The chromatograph (6850 Network GC System, Agilent Technologies) was equipped with a flame ionization detector. Injector and detector temperatures were kept constant at 250 °C (conditions: anisole was used as an internal standard, initial column temperature 40 °C; column final temperature 200 °C). The conversion was calculated by detecting the decrease of the monomer peak area relative to the internal standard peak area. The degree of polymerization (DP) and subsequent $M_{n,GC}$ was calculated based on the initial molar ratio of monomer to initiator and conversions obtained from GC. The number average molecular weights of copolymers were calculated by a summary of the weight of PDMAEMA block and polyester macroinitiator, which chains lengths were calculated on the basis of end groups on ^{1}H NMR spectrum.*Attenuated total reflectance-Fourier transform infrared spectroscopy (ATR-FTIR)*

ATR-FTIR analysis was carried out with a NICOLET 670 spectrophotometer at room temperature by attenuated total reflection (ATR) method. Spectra were recorded at 16 scans per spectrum and 4 cm^{-1} resolution in the range of 4000–400 cm^{-1}.

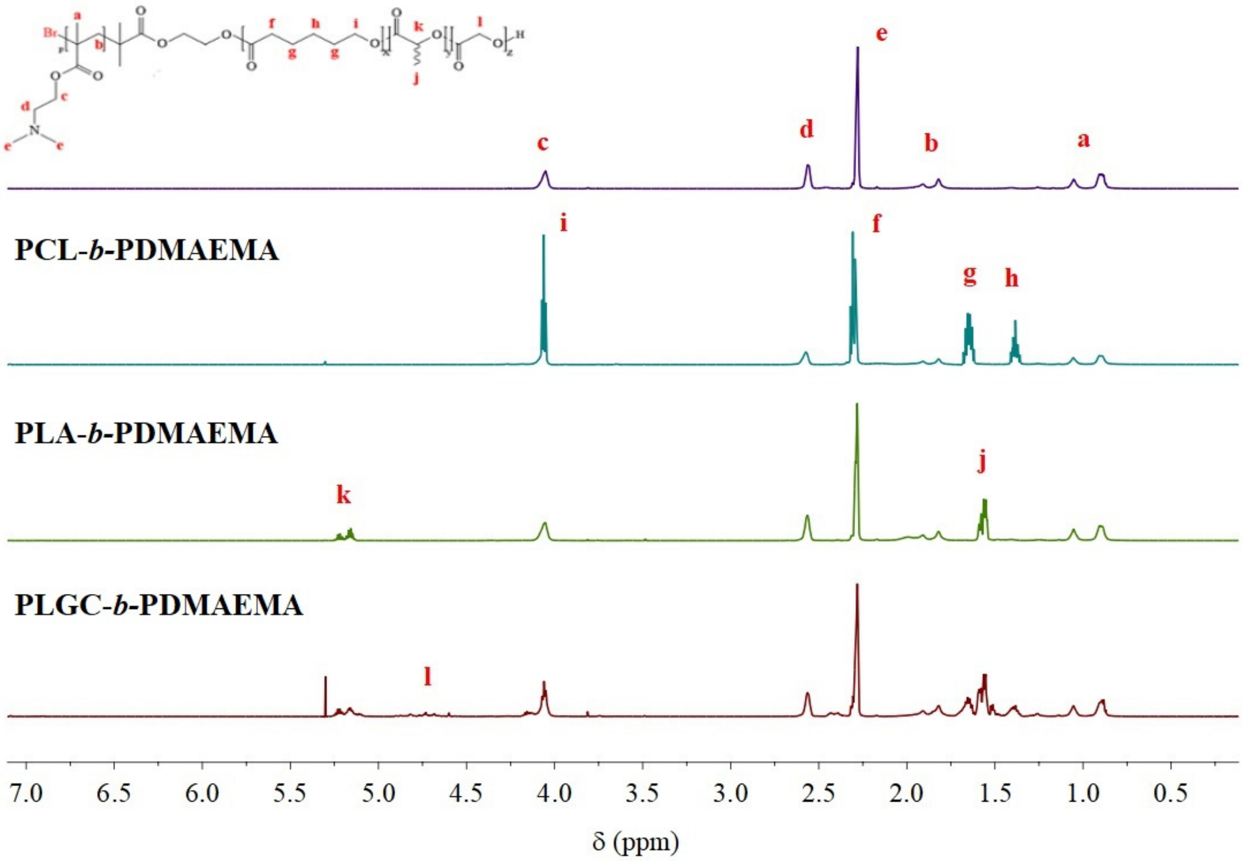

Figure S1. ^{1}H NMR (600 MHz, $CDCl_3$) spectra of obtained copolymers before degradation.

^{1}H NMR (600 MHz, $CDCl_3$, δ) for PDMAEMA: 4.10–4.00 (2H, s, $-O-CH_2-$); 2.61–2.53 (2H, s, $-CH_2-N(CH_3)_2$); 2.35–2.25 (6H, s, $-N(CH_3)_2$); 2.10–1.75 (2H, m, $-CH_2-$); 1.15–0.80 (3H, m, $-CH_3$)

^{1}H NMR (600 MHz, $CDCl_3$, δ) for PCL-*b*-PDMAEMA: 4.10–4.00 (2H, t, $-O-CH_2-$); 3.79–3.76 (2H, $-CH_2-OH$); 2.61–2.53 (2H,s, $-CH_2-N(CH_3)_2$); 2.35–2.25 (2H, m, $-C(O)-CH_2-$); 1.68–1.60 (4H, m, $-C(O)-CH_2-\underline{CH_2}-CH_2-\underline{CH_2}-$); 1.41–1.34 (2H, m, $-C(O)-CH_2-CH_2-\underline{CH_2}-CH_2-$); 1.15–0.80 (3H, m, $-CH_3$).

^{1}H NMR (600 MHz, $CDCl_3$, δ) for PLA-*b*-PDMAEMA: 5.28–5.10 (1H, m, $-\underline{CH}(CH_3)-O-$); 4.44–4.22 (1H, q, $-\underline{CH}(CH_3)-OH$); 4.10–4.00 (2H, t, $-O-CH_2-$); 2.61–2.53 (2H, s, $-CH_2-N(CH_3)_2$); 2.35–2.25 (6H, s, $-N(CH_3)_2$); 2.10–1.75 (2H, m, $-CH_2-$); 1.60–1.51 (3H, m, $-CH_3$); 1.15–0.80 (3H, m, $-CH_3$).

^{1}H NMR (600 MHz, $CDCl_3$, δ) for PLGC-*b*-PDMAEMA: 5.28–5.10 (1H, m, $-\underline{CH}(CH_3)-O-$); 4.92–4.56 (2H, m, $-CH_2-O-$); 4.44–4.22 (1H, q, $-\underline{CH}(CH_3)-OH$); 4.10–4.00 (2H, t, $-O-CH_2-$); 3.79–3.76 (2H, m, $-CH_2-OH$); 2.61–2.53 (2H, s, $-CH_2-N(CH_3)_2$); 2.35–2.25 (6H, s, $-N(CH_3)_2$); 2.10–1.75 (2H, m, $-CH_2-$); 1.68–1.60 (4H, m, $-C(O)-CH_2-\underline{CH_2}-CH_2-\underline{CH_2}-$); 1.60–1.51 (3H, m, $-CH_3$); 1.41–1.34 (2H, m, $-C(O)-CH_2-CH_2-\underline{CH_2}-CH_2-$); 1.15–0.80 (3H, m, $-CH_3$)

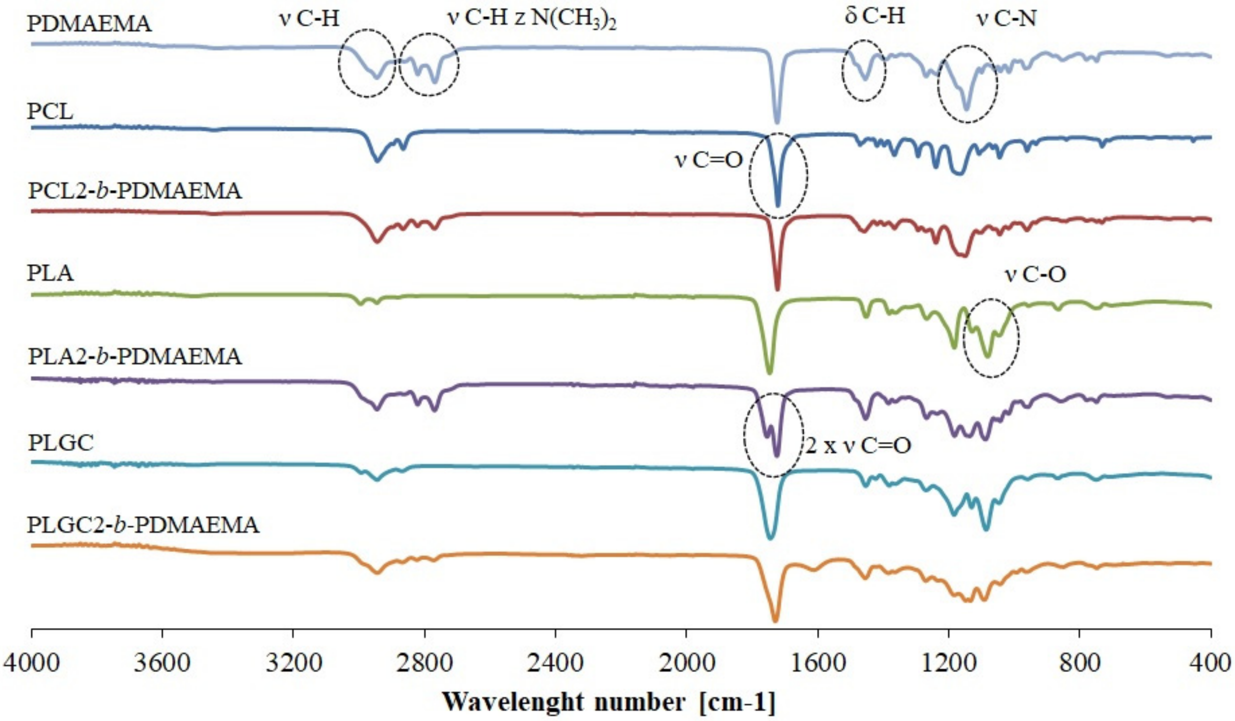

Figure S2. ATR-FTIR spectra of obtained polymers before degradation.
